# Diabetes and Cardiovascular Risk in Renal Transplant Patients

**DOI:** 10.3390/ijms22073422

**Published:** 2021-03-26

**Authors:** Jacek Rysz, Beata Franczyk, Maciej Radek, Aleksandra Ciałkowska-Rysz, Anna Gluba-Brzózka

**Affiliations:** 1 Department of Nephrology, Hypertension and Family Medicine, Medical University of Lodz, 90-549 Lodz, Poland; jacek.rysz@umed.lodz.pl (J.R.); beata.franczyk-skora@umed.lodz.pl (B.F.); 2 Department of Neurosurgery, Surgery of Spine and Peripheral Nerves, Medical University of Lodz, 90-549 Lodz, Poland; maciej.radek@umed.lodz.pl; 3 Palliative Medicine Unit, Department of Oncology, Medical University of Lodz, 90-549 Lodz, Poland; aleksandra.cialkowska-rysz@umed.lodz.pl

**Keywords:** end-stage renal diseases, prediabetes, post-transplant diabetes mellitus, insulin resistance, transplantation, treatment, guidelines

## Abstract

End-stage kidney disease (ESKD) is a main public health problem, the prevalence of which is continuously increasing worldwide. Due to adverse effects of renal replacement therapies, kidney transplantation seems to be the optimal form of therapy with significantly improved survival, quality of life and diminished overall costs compared with dialysis. However, post-transplant patients frequently suffer from post-transplant diabetes mellitus (PTDM) which an important risk factor for cardiovascular and cardiovascular-related deaths after transplantation. The management of post-transplant diabetes resembles that of diabetes in the general population as it is based on strict glycemic control as well as screening and treatment of common complications. Lifestyle interventions accompanied by the tailoring of immunosuppressive regimen may be of key importance to mitigate PTDM-associated complications in kidney transplant patients. More transplant-specific approach can include the exchange of tacrolimus with an alternative immunosuppressant (cyclosporine or mammalian target of rapamycin (mTOR) inhibitor), the decrease or cessation of corticosteroid therapy and caution in the prescribing of diuretics since they are independently connected with post-transplant diabetes. Early identification of high-risk patients for cardiovascular diseases enables timely introduction of appropriate therapeutic strategy and results in higher survival rates for patients with a transplanted kidney.

## 1. Introduction

End-stage kidney disease (ESKD) is a main public health problem, the prevalence of which is continuously increasing worldwide [1]. Due to the adverse effects of renal replacement therapies, kidney transplantation seems to be the optimal form of therapy with significantly improved survival, quality of life, and diminished overall costs compared with dialysis [2,3,4]. However, it does not mean that transplant patients are devoid of elevated mortality rates compared with the general population [5]. Such increased mortality is associated with a high rate of cardiovascular and cardiovascular-related deaths after the transplantation, which constitutes over 50% of all deaths in this group [6,7]. Numerous studies have also indicated that gender issues in transplantation are highly important as they not only exert an impact on graft functioning and the risk of rejections but also on the post-transplant diabetes mellitus (PTDM) development hazard. Nowadays, donors frequently cannot be matched by gender due to the not sufficient number of donors and high demand for transplantations. However, female-mismatch patients (male donor/female recipient) were found to have more episodes of acute rejection and more rehospitalizations [8,9]. Such finding could be ascribed not only to weight-mismatch or size-mismatch of an organ but also hormonal discrepancies. Several studies have demonstrated that male renal allografts may function better over time than grafts from female donors, while those from female donors show worse renal allograft survival [10,11,12,13]. According to studies, recipients of male kidneys are less likely to require antirejection therapies beyond their baseline immunosuppression and their renal function is significantly improved after transplantation [13]. In turn, female kidney recipients seem to be more likely to have acute rejection episodes (possibly as a result of sensitization after pregnancy), but less likely to develop chronic graft rejection [10,14]. It has also been suggested that benefits related to the transplantation of kidney from a male donor are associated with the fact that organs from males have greater number of nephrons or mass than female kidneys, however, this theory of “nephron under-dosing” has not been confirmed. There are also numerous pieces of evidence that the complex impact of androgens and estrogens on the functioning of many cells as well as immunologic differences between men and women may contribute to worse renal graft outcomes with female donors [10,13].

Post-transplant diabetes mellitus (PTDM) is an important risk factor for this enhanced risk. In the study of stable kidney transplant patients, 43% of participants had new impaired glucose metabolism developed within 6 months from the transplantation [15]. Numerous studies confirmed higher rates of cardiovascular disease occurrence, cardiovascular death and all-cause mortality in post-transplant patients [16,17]. Additionally, other forms of impaired glucose metabolism after transplantation seem to be associated with increased mortality [18].

The selection of articles for this literature review was based on a PubMed search (terms: “PTDM” + “risk factors” + “pathomechanism” + “cardiovascular risk” were applied). We tried to focus on those studies which were performed in large groups or which results were, in our opinion, particularly interesting.

## 2. Prediabetes and Post-Transplant Diabetes Mellitus (PTDM)—Diagnosis and Prevalence

Prediabetes and post-transplant diabetes are frequently occurring in renal transplant patients. According to the definition, prediabetes is an intermediate metabolic state between normoglycemia and diabetes [19]. The diagnosis of prediabetes is made on the basis of the presence of impaired fasting glucose (IFG), impaired glucose tolerance (IGT) in ordinary oral glucose tolerance test and glycated hemoglobin A1c (HbA1c) concentration between 5.7% and 6.4% [20]. This state does not fulfill the criteria of diabetes mellitus (DM), however, it is an independent risk factor for the progression to DM or post-transplantation diabetes mellitus (PTDM) [21]. The mechanisms of prediabetes pathogenesis remain not fully resolved. It has been suggested that impaired fasting glycemia may be associated with peripheral insulin resistance, while impaired glucose tolerance (IGT) with impaired β-cell function [22,23].

PTDM is defined as “newly diagnosed diabetes mellitus in the post-transplant setting (irrespective of timing or whether it was present but undetected prior to transplantation or not)” [16,24]. For the first time, post-transplantation diabetes mellitus was described in kidney transplant recipients in 1964 [25,26]. Through the years, the nomenclature of this disease has changed several times. In 2014, the International Expert Panel comprising transplant nephrologists, diabetologists, and clinical scientists recommended the replacement of NODAT (new-onset diabetes after transplantation) term with PTDM as a result of a high prevalence of undiagnosed pretransplant diabetes mellitus [16].

The diagnosis of post-transplant diabetes has been challenging for a long time. The first clear diagnostic criteria were introduced in 2003 by the American Diabetes Association and the World Health Organization (WHO) [17]. These guidelines have been updated in 2010 and according to them the following criteria must be met to diagnose post-transplant diabetes: “symptoms of diabetes and a non-fasting plasma glucose (PG) of > 200 mg/dL (11.1 mmol/L), fasting PG of > 126 mg/dL (7.0 mmol/L), PG > 200 mg/dL (11.1 mmol/L) 2 h following an oral glucose tolerance test and HbA1C > 6.5% (48 mmol/mol) [27].

According to estimations, prediabetes and post-transplant diabetes mellitus can affect about 10–30% of renal transplant patients [19,28,29]. Prospective studies utilizing contemporary consensus-based diagnostic criteria (e.g., repeated oral glucose tests) have confirmed the occurrence of PTDM in about 30% of renal transplant recipients and prediabetes in another 20% of this population [28]. The rates of PTDM may increase with time and reach 27% within 3 months and 30% after 36 months from the transplantation [28]. The highest rate of PTDM occurrence (83.7%) is observed within the first year [30]. However, due to the early reversibility rate of IFG or IGT, the diagnosis of prediabetes and PTDM in stable clinical conditions should be performed after the first 12 months from transplantation [19].

## 3. Prediabetes and Post-Transplant Diabetes Mellitus (PTDM)—Risk Factors and Pathophysiology

The pathomechanism of PTDM is not fully resolved. β-cell dysfunction is believed to be the vital mechanism involved in the development of PTDM due to the modification in insulin secretion [31]. Enhanced insulin resistance and predominantly decreased insulin secretion are considered to be the underlying causes of PTDM [15,32]. It remains unknown, whether the risk of progression to PTDM differs between patients with IFG and IGT, however, such knowledge would allow for further risk stratification [20]. The PTDM develops in the consequence of the presence of predisposing factors (which are similar to type 2 diabetes mellitus) as well as risk factors related to the transplantation, among which the most important are the following: metabolic adverse effects of immunosuppressive therapy with calcineurin inhibitors, mammalian target of rapamycin inhibitors (mTORi), and corticosteroids, infections with cytomegalovirus (CMV) and hepatitis C virus after the transplantation and hypomagnesemia [33,34,35,36]. According to studies, PTDM is nearly four times more frequent in anti-HCV-positive patients compared to uninfected individuals due to the fact that the hepatitis C virus stimulates the process of apoptosis-like death of pancreatic β -cells via the caspase 3-dependent pathway [37,38,39]. As CMV infection has been confirmed to be a risk factor for increasing incidence of PTDM, the prophylaxis against CMV infection after kidney transplantation is strongly recommended [40]. The impact of CMV on diminishing insulin secretion may involve direct β -cell damage by CMV and apoptosis or their destruction by infiltrative leukocytes or it is the result of the induction of proinflammatory cytokines. According to studies, allograft-associated factors, such as graft type have an impact on the development of PTDM [41]. Deceased donor allografts express higher levels of proinflammatory cytokines than allografts from living donors and it seems that the presence of such a proinflammatory state predisposes to the PTDM. Indeed, in the recipients of deceased donor grafts higher prevalence of PTDM is observed compared to living donor grafts (hazard ratio (HR) 3.3 (1.46–7.52), *p* = 0.004 risk) [42].

According to Cosio et al. [43], the increasing incidence of PTDM is related to the greatly improved bioavailability of calcineurin inhibitors (CNIs) and thus increased exposure to their diabetogenic properties as well as the change in recipient characteristics, particularly the increased body weight at the time of transplantation. Immunosuppressive drugs (calcineurin inhibitors, corticosteroids and mTORi) have been confirmed to exert adverse metabolic effects [33]. Calcineurin inhibitors (CNIs) have been shown to induce PTDM through multiple mechanisms, including the diminishing of insulin secretion and due to direct toxic effects on pancreatic β- cells [37]. The analysis of pancreatic histology sections confirmed that the administration of CNIs was associated with enhanced islet cell apoptosis and reduced β-cell mass [44,45]. This effect is related to the fact that calcineurin activates the transcription factors nuclear factor of activated T-cells (NFAT) which stimulates IRS2 transcription and cAMP response element-binding protein (CREB) mediating proliferative effects of glucagon-like peptide (GLP-1) and, therefore, it affects the survival of β-cells in the pancreas [41]. In turn, CNIs downregulate IRS2 expression via the inhibition of both NFAT and CREB [46]. Experimental studies confirmed that calcineurin inhibition decreased Akt phosphorylation in murine and human islets [46]. Some studies suggested that therapeutic levels of cyclosporine and tacrolimus could hinder glucose uptake into adipose cells via the stimulation of endocytosis of glucose transporters type 4 (GLUT 4) from the cell surface, while others failed to demonstrate strong impact of CNIs on insulin sensitivity [47,48].

The negative effects of therapy with corticosteroids involve the impairment of insulin secretion, the aggravation of insulin resistance related to higher rates of gluconeogenesis in the liver as well as indirect impact on weight gain, the rise in lipolysis-induced dyslipidemia, and the decrease in muscle mass, glycogen synthesis and glucose uptake in skeletal muscle cells [35,46,49]. However, the resignation from such treatment may improve insulin sensitivity but it also significantly enhances the risk of acute graft rejection, and worsens proteinuria and glomerulonephritis recurrence [50,51,52]. Tacrolimus-related adverse diabetogenic effects include the reduction in insulin gene transcription, β-cell apoptosis, and the reduction in insulin exocytosis [53]. Therefore, it seems that the adjustment of its dose may improve pancreatic β-cell function [54].

Additionally, mTOR inhibitors (sirolimus) have been found to be associated with a higher risk for PTDM as a result of sirolimus-induced hyperglycemia caused by compromised insulin-mediated suppression of hepatic glucose production, the accumulation of ectopic triglycerides and subsequent insulin resistance, as well as direct pancreatic β-cell toxicity [49]. mTOR inhibitors act on the insulin receptor-IRS-PI3K-Akt pathway; the activation of Akt and subsequent stimulation of protein synthesis require the activation of the mTOR-containing complex (mTORC1). The binding of sirolimus to mTOR results in the stimulation of the phosphorylation and the repression of IRS-1 leading to the inhibition of P13K/Akt signaling [55]. Sirolimus has been demonstrated to induce hyperinsulinemia, glucose intolerance, and hypertriglyceridemia as the result of enhanced hepatic gluconeogenesis and diminished stimulated glucose uptake in skeletal muscle [33,56,57]. The impact of everolimus on islet cell function is weaker compared to sirolimus, however, the rejection rates, in this case, are higher and some studies indicated that its administration brought no benefits in terms of the incidence of PTDM [58,59]. Moreover, similarly to CNIs, mTORi could promote apoptosis of pancreatic islet cells in vitro [60]. Besides, sirolimus has been found to impair pancreatic ductal proliferation and diminish ductal cell numbers in a culture which could translate into decreased glucose-stimulated insulin secretion [61]. It seems that the impact of mTOR inhibitors on insulin signaling is much more important than on insulin secretion. According to a large, retrospective study, treatment with sirolimus is an independent and vital risk factor for the development of PTDM, the impact of which is as strong as that of obesity or older age [62].

Additionally, the use of glucocorticoids was found to be associated with hyperglycemia resulting from the increase in glucose resistance, the reduction in insulin secretion, and the induction of β-cells apoptosis leading to reduced expression of glucose transporter 2 and glucokinase [63]. The effect is dose-dependent.

Among other modifiable risk factors of PTDM, there are the following: obesity, metabolic syndrome, and physical activity. While, the nonmodifiable include family history of DM, age, the presence of autosomal-dominant polycystic kidney disease (ADPKD), African American and Hispanic origin as well as some human leukocyte antigen (HLA) genotypes, including *HLA-B27*, *HLA-DR3*, and *HLA-A3* [17,33,34,64,65]. Higher PTDM risk is observed in transplant patients of Hispanic and Caucasian and African American origin [66,67]. The results concerning the relationship between a family history of diabetes and PTDM are conflicting. Some studies failed to find any association [30,68], while other indicated that diabetes in first-degree relatives was an independent risk factor of glucose metabolism disorders or PTDM after kidney transplantation [69,70]. Gene polymorphisms within a leptin receptor and a cytochrome CYP24A1 have also been suggested as emerging risk factors for PTDM, however, further studies are required to assess their impact [71,72].

It has been suggested that genes participating in the regulation of lipid homeostasis and carbohydrate metabolism could be associated with the development of PTDM. Yang et al. [73] observed that in kidney transplant patients of Hispanic ethnicity 2 alleles of the *HNF-4A* gene encoding transcription factor (*rs2144908* and *rs1884614*) were significantly associated with PTDM. Adiponectin and leptin have been demonstrated to be implicated in the regulation of insulin secretion, glucose and lipid metabolism. For example, *LEP rs2167270* gene polymorphism was shown to be considerably associated with enhanced risk of PTDM [74]. Moreover, some studies suggested that mannose-binding lectin 2 (MBL2), which is a vital recognition molecule of the lectin pathway of complement activation, could be involved in the pathogenesis of PTDM since it could play an important role in noninfectious inflammatory damage following organ transplantation [75,76]. The results of the study conducted by Guad et al. [77] demonstrated that indeed AG heterozygous variant of the *MBL2* gene (*rs2232365*) was associated with an elevated risk of PTDM compared to AA variant. It seems that genetic polymorphisms within this gene may affect insulin secretion from the pancreas. Other groups of genes assessed in their relation to PTDM are interleukins (ILs) and inflammation-related factors. Inflammatory chemokines and cytokines have been shown to be involved in peripheral insulin action and insulin secretion [78]. ILs and other molecules secreted by T cells mediate inflammation via the promotion of the production of inflammatory cytokines (TNF-α, IL-1B, and IL-6) [78]. G allele at position 2174 of the IL-6 gene promoter has been shown to be associated with the risk of PTDM among overweight subjects [79]. Additionally, variations of interleukin (IL)-7R, IL-17E, IL-17R, and IL-17RB were found to influence this risk, which may imply that the inflammation of islet β-cells might be of fundamental importance in the pathogenesis of PTDM in renal transplantation recipients [80]. Bamoulid et al. [79] found that in GG homozygotes (IL-6-174 SNP) the risk of PTDM was significantly higher, however, this effect was restricted mostly to overweight patients. Moreover, they demonstrated the link between G allele and serum IL-6 levels and lower insulin sensitivity in the GG carriers than in the CC carriers (2.15 ± 2 versus 1.32 ± 1.03; *p* = 0.03). In turn, Weng et al. [81] indicated a decreased risk of PTDM development in carriers of IL-6 G/G genotype. The analysis of the panel of 18 SNPs within 10 genes of IL or their receptors revealed that 11 of them (61.1%) were significantly associated with PTDM development after adjusting for sex, age and tacrolimus usage, which may confirm the thesis that inflammation of islet β cells is vital in the pathogenesis of PTDM in renal transplantation recipients [80]. These polymorphisms were in IL-1B (*rs3136558*), IL-2 (*rs2069762*), IL-4 (*rs2243250, rs2070874*), IL-7R (*rs1494558, rs2172749*), IL-17RE (*rs1124053*), IL-17R (*rs2229151, rs4819554*), and IL-17RB (*rs1043261, rs1025689*). The last four SNPs have been previously linked with type 1 diabetes mellitus [80].

Meta-analysis of genetic association studies found the relationship between interleukin-7 (IL7) (*rs1494558*), potassium voltage-gated channel subfamily Q member 1 (KCNQ1) (*rs2237892*), and transcription factor 7 like 2 (TCF7L2) (*rs7903146*) and PTDM development [82]. A genomic-wide association study (GWAS) indicates that TCF7L2, which belongs to T-cell transcription factor family controlling cell proliferation and differentiation as well as regulating pancreas development, maturation and islet function, is significantly associated with type 2 diabetes [83]. Some studies showed the relationship between the presence of T allele *TCF7L2 rs7903146* and enhanced protein expression, compromised insulin secretion, impaired incretin effects and hepatic insulin resistance, however, other studies did not support these data [73,84,85,86,87]. Large meta-analysis confirmed that *rs7903146* T variant increased the risk of PTDM in an allele dose-dependent manner [82].

Chen et al. [88] indicated that polymorphisms in the *NFATc4* gene might confer certain protection or predisposition for PTDM. NFAT genes code transcription factor of the nuclear factor of activated T cells (NFAT) which regulates immune activation and insulin production. Calcineurin inhibitors (CNIs) have been shown to activate NFAT. According to the aforementioned authors, T allele (*rs10141896*) was associated with a lower cumulative incidence of PTDM (*p* = 0.02). They demonstrated that CNI-treated recipients with one of the five dominant *NFATc4* haplotypes, T-T-T-T-G, had a reduced adjusted risk for PTDM (hazard ratio (HR): 0.45; 95% confidence interval (Cl): 0.19–1.01). In turn, in patients homozygous for the C-C-C-G-G haplotype enhanced risk (HR: 2.13; 95% Cl: 1.01–4.46) for PTDM was reported. Additionally, polymorphisms in C-C motif chemokine ligand 2 (CCL2) gene which codes an inflammatory or inducible chemokine and C-C motif chemokine ligand 5 (CCL5) gene which is a target of NF- B activity, have been suggested to alter the risk of post-transplant diabetes mellitus development. Dabrowska-Zamojcin et al. [89] found that the number of *CCL2 rs1024611* G alleles (HR 1.65; 95%CI 1.08–2.53; *p* = 0.021) was significantly positively associated with the risk of PTDM onset in patients treated with tacrolimus or cyclosporine, irrespectively of organ recipients’ sex, age and BMI. In the Korean population polymorphisms within *CCL5* gene (*rs2107538, rs2280789* and *rs3817655*) (TCA haplotype) were found to be significantly associated with increased risk of PTDM [90].

In patients without diagnosed diabetes before renal transplantation, single nucleotide polymorphism (SNP) within angiotensinogen (AGT) (*rs4762*) seem to increase the risk of PTDM development in the dominant models (*p* = 0.03) after adjusting for age and tacrolimus usage, however, exact molecular mechanism of this relationship requires clarification [91]. In addition, polymorphism within a gene involved in oxidative stress (*GPX1*, SNP *rs1050450*) was associated with an increased risk of PTDM [92]. Functional polymorphisms in this gene have been previously demonstrated to increase the risk of cardiovascular and peripheral vascular diseases in type 2 diabetic patients [93,94]. Finally, the SNPs in genes involved in tacrolimus metabolism, including peroxisome proliferator-activated receptor α (PPARα) and P450 oxidoreductase (POR) have drawn attention. These two genes participate in the regulation of energy uptake, lipid and carbohydrate metabolism. Three polymorphisms, a coding POR variant (*rs1057868*) and two SNPs in PPARα (*rs4823613* and *rs4253728*) were found to enhance the risk of PTDM [95]. Patients carrying multiple predisposing SNPs show a greater risk of PTDM [41]. The number of SNP in genes associated with PTDM is so large that it is impossible to mention all of them in this review.

Metabolic alterations occurring before the transplantation, including glucose metabolism impairment and increase in BMI (Body Mass Index) seem to play important role in the enhancement of PTDM risk [96]. Hypoglycemia before the transplantation, mirroring the presence of early insulin resistance or insulin secretion deficiency, eventually contributes to PTDM [97]. The presence of prediabetes (IGT or IFG), with fasting plasma glucose <7 mmol/L or fasting plasma glucose ≥6.1 and <7 mmol/L and 2-h plasma glucose after an oral glucose tolerance test (OGTT) ≥7.8 and <11.1 mmol/L are risk factors for the future development of diabetes, not only in post-transplant patients but also in general population [16]. According to studies, 15% of transplant patients with impaired glucose tolerance will develop PTDM after 1 year and another 27% of them over 6 years [98,99]. Additionally, perioperative hyperglycemia associated with a stress reaction to the surgery itself (the release of catecholamines), the administration of corticosteroids as well as the renal function, increases the risk of PTDM [37].

The age higher than 45 years old increases the risk of PTDM 2.2 times compared to transplant patients aged 18–44 years [66]. According to studies, higher age is related to diminished insulin secretion which mirrors the typical aging process and the apoptosis of β-cells. It has been indicated that among patients over the age of 48 years on a waiting list, the relative risk of prediabetes was 2.5-fold higher than in younger patients [20]. Additionally, higher BMI strongly influenced insulin sensitivity. Abdominal circumference of more than 94 cm at the time of renal transplantation was shown to be a vital independent PTDM risk factor in men, while in women BMI value at the time of renal transplantation was more important [100]. Obesity at transplantation has been confirmed to be an independent risk factor for PTDM [69,101,102]. A large study of renal transplant recipients showed that the risk of PTDM was enhanced in overweight patients (BMI > 25 kg/m^2^), and became obvious in obese subjects (BMI > 30 kg/m^2^) [102]. The relative risk for PTDM in obese patients was 1.73 (95% CI 1.57–1.90, *p* < 0.0001). Another study indicated that PTDM risk increased linearly for every 1 kg above 45 kg [103]. A greater PTDM risk in overweight patients may be the consequence of a chronic inflammatory state related to excessive fat which stimulates macrophage recruitment to adipocytes and the release of proinflammatory adipokines leading ultimately to the downregulation of insulin signaling [33,104,105]. Moreover, adipose tissue produces tumor necrosis factor-alpha (TNFα), the activation of which is associated with insulin resistance via the reduced expression of insulin-sensitive glucose transporters [106]. Rodrigo et al. [106] found that every 1 μg/mL decrease in adiponectin enhanced the risk of developing PTDM by 13%. In addition, proteinuria (low-grade proteinuria and very low-grade (<0.3 g/day)) which develops within 3–6 months from the transplantation was shown to be a strong independent risk factor for PTDM [107].

The disturbances in lipid profile, especially the rise in TG/HDL (triglyceride/high-density lipoprotein cholesterol) ratio (above 3.5) also increase the incidence of diabetes mellitus, as they are associated with the worsening of glucose homeostasis and poor glycemic control in women with type 2 DM [108]. According to de Lucena et al. [33], higher ratios of TG/HDL in post-transplant patients may be utilized as a surrogate marker of insulin resistance. Bayés et al. [109] demonstrated that low levels of adiponectin, increased levels of C-reactive protein and triglycerides, and high BMI before transplant were predictors of PTDM.

According to studies, also tacrolimus-related electrolyte abnormalities could raise the risk of PTDM [41]. Hypomagnesemia, which is frequently observed after tacrolimus therapy, has been demonstrated to be an independent risk factor for insulin resistance and hyperglycemia [110]. Van Laecke et al. [111] revealed that an Mg level <1.9 mg/dL compared to higher values was associated with the more accelerated development of PTDM in renal transplant recipients (log-rank *p* < 0.001). Finally, the hazard ratio for the development of PTDM is higher in patients with acute rejection after transplantation (HR 3.7), however, this finding may be associated also with antirejection therapy involving high-dose steroid and increased CNIs [111].

Experimental studies have shown that gender issues in transplantation are highly important as they not only exert an impact on the PTDM development hazard. Some risk factors are equally important in men and women, while others seem to be more relevant in one sex. The study of renal transplant patients revealed that age at the time of transplantation (KT) >60 years and hypovitaminosis D at the time of KT (<20 μg/L) were independent risk factors for PTDM in both sexes, while the strong relationship between a waist circumference at the time of KT > 94 cm, HOMA-IR > 2, C-peptide at the time of KT > 5 ng/mL, and triacylglycerols at the time of KT > 1.7 mmol/L and PTDM was observed only in men [100]. In turn, in women, BMI at the time of KT > 30 kg/m^2^ and menopause at the time of KT were dominant factors. Moreover, the authors found that women displayed pancreas β cell dysfunction, whereas insulin resistance and metabolic syndrome were principal in men [100].

Early identification of patients at risk of PTDM may enable the determination of appropriate strategies (e.g., lifestyle modification and modification of pharmacological treatment) to reduce its occurrence [112,113]. In contrast to PTDM, which is diagnosed and treated early, prediabetes is not looked for in renal transplant patients [19]. It seems strange since the identification and treatment of prediabetes may enable the reduction of the burden of diabetes and cardiovascular disease in this population.

Despite the fact that PTDM and diabetes mellitus type 2 share some predisposing factors and many characteristics, the prevention and treatment of these two disorders are often dissimilar [33].

Hyperglycemia undoubtedly exerts a serious adverse impact on post-transplant outcomes [114]. The development of PTDM appears to be an important risk factor for cardiovascular morbidity and mortality after transplantation. Abundant studies have indicated the relation between PTDM and enhanced risk of cardiovascular events [6,115]. Table 1 presents the summary of the results of studies concerning PTDM risk factors and involved pathomechanisms accompanied by our estimation of the strength of evidence.

## 4. Possible Biomarkers in PTDM

The review of available most recent publication has not revealed any predictive or prognostic biomarkers of PTDM showing high sensitivity and specificity. Some articles indicate the association between the inflammation of the pancreatic β-cells and PTDM as well as the link between enhanced innate immune system activity and development of PTDM [80,116,117]. Others suggest the involvement of tumor necrosis factor (TNF). Elevated levels of high sensitivity interleukin IL-6 were observed in PTDM patients by the team of Cieniawski and colleagues [118]. In turn, Heldal et al. [116] demonstrated a strong relationship between four inflammation-related parameters (sTNFR1, EPCR, PTX3, and MIF) and plasma glucose levels 2 h after an OGTT in patients, 10 weeks after kidney transplantation. This association remained significant after the adjustment for age, BMI, graft function, insulin levels, a calcineurin inhibitor, and prednisolone dose. Their finding may imply the role of an inflammatory state in the development of PTDM. As it has been mentioned in previous sections, decreased insulin secretion from the pancreatic islets and enhanced peripheral insulin resistance are observed in the course of PTDM [15,116,119]. The results of the Heldal et al. [116] study showing the association between markers of inflammation and impaired 2-h plasma glucose and the results of studies in which the presence of peripheral insulin resistance in T2DM was related to low-grade inflammation suggest similar mechanisms in both diseases. Therefore, it has been hypothesized that in renal transplant recipients, inflammatory microenvironment could promote the development of peripheral insulin resistance and may lead to impaired insulin secretion in pancreatic β-cells [80,117,120].

A large prospective cohort study of stable renal transplant recipients demonstrated a direct link between plasma malondialdehyde (MDA) and plasma glucose concentration [121]. Moreover, this study indicated that plasma MDA level inversely correlated with long-term risk of PTDM, even after the adjustment for potential confounders, such as BMI, baseline glucose concentration, and immunosuppressive therapy. This finding may suggest that also oxidative status plays an important role in glucose homeostasis.

Most studies examining genes related to PTDM focus on those previously associated with type 2 diabetes due to the fact that these two diseases share similar mechanisms controlling insulin production and maintenance of stable glucose levels, including insulin resistance and insulin hypo-secretion and analogous risk factors (advanced age, family history of diabetes and non-white ethnicity) [122]. One of analyses of genetic variants associated with PTDM in kidney transplant patients showed that TCF7L2 rs7903146, CDKAL1 rs10946398 and KCNQ1 rs2237892 were significantly associated with PTDM [82]. Polymorphisms within transcription factor 7-like-2 (TCF7L2) have been previously linked with DM type 2. The T allele was identified as a diabetes risk factor, however, the mechanism via which TCF7L2 influenced the risk of diabetes has not yet been completely resolved. It has been suggested to exert an impact on blood glucose homeostasis via altering levels of glucagon-like peptide 1 in the gut or to reduce insulin secretion via the pancreatic β, adipose, or liver cells [123]. In turn, cyclin-dependent kinase 5 regulatory subunit associated protein 1 like 1 (CDKAL1) has been found to be associated with impaired insulin secretion as it regulates CDK5 protein involved in the promotion of insulin production as well as the development of DM type 2 [124]. It was suggested that CDKAL1-related overexpression of CDK5 might increase the risk of DMT2 and NODAT by impairing insulin production [82]. Additionally, KCNQ1 was found to be a well-known risk factor for type 2 diabetes mellitus. The protein encoded by KCNQ1 is expressed in pancreatic islet cells (not only) and together with KCNE proteins they form voltage charged potassium channels found in the membranes. As pancreatic βcell survival rate is believed to be influenced by these potassium channels, their dysfunction could alter cell membrane potential and contribute to the development of T2D or NODAT [82]. Schultz et al. [125] suggested that homozygous carriers of C allele (rs2237892) showed impaired baseline insulin secretion.

MicroRNA (miRNA) have gained a lot of attention due to the fact that they are potential sensitive biomarkers in numerous human diseases and tissue injury [37]. They play an essential role in the modulation of gene expression as they bind to 3’ untranslated region of miRNA of protein-coding genes to downregulate their expression thus affecting almost every key cellular function [126]. miRNAs are short (19–23 nucleotides) non-coding RNA produced endogenously [37]. We found no available studies analyzing the role of miRNA in PTDM, however, the research of the repertoire of miRNAs involved in the development and progression of diabetes mellitus is better studied and it could be hypothesized that some alterations in the level of miRNA may be similar in DM and PTDM. Huang et al. [127] indicated that the level of miR-155 and miR-146a was over five times higher in diabetic samples compared to controls and they strongly correlated with serum creatinine levels. In a rat model, the induction of the aforementioned miRNA was found to increase gradually. According to some studies, also miR-126 can be utilized as a biomarker for prediabetes and DM as its levels were considerably diminished in patients with impaired fasting glucose, impaired glucose tolerance or with DM compared to healthy controls [128]. The pathogenesis of diabetic nephropathy and insulin resistance was found to be associated with the deregulation of miR-29. Patients with albuminuria demonstrated much higher levels of urinary miR-29a than those with normal albuminuria [129]. Celen et al. [130] suggested that miRNA biogenesis pathways might be affected by immunosuppressive treatment. They found that miRNA biogenesis components could serve as potential biomarkers indicative of graft outcome. Gene expression of these components was demonstrated to be considerably decreased after transplantation and probably even more reduced in patients undergoing chronic rejection [130]. In turn, Ulbing et al. [131] observed that the systemic expression of miR-223-3p and miR-93-5p was significantly diminished during later stages of CKD, however, kidney transplantation fully reversed this effect. These two miRNAs are strongly associated not only with CKD stages and kidney function but also with inflammatory state parameters and indices of glucose metabolism. Zampetaki et al. [132] revealed reduced plasma levels of miR-20b, miR-21, miR-24, miR-15a, miR-126, miR-191, miR-197, miR-223, miR-320, and miR-486 in prevalent DM. Moreover, they indicated that decreased miR-15a, miR-29b, miR-126, miR-223, and elevated miR-28-3p levels preceded the manifestation of diabetes.

## 5. Cardiovascular Risk and Morbidity of Renal Transplant Patients

Risk factors for the development of cardiovascular diseases in kidney transplant patients are more abundant compared to the general population and comprise traditional factors (diabetes mellitus, hypertension, hyperlipidemia), factors related to decreased glomerular filtration (anemia, hyperhomocysteinemia) as well transplantation-specific factors (immunosuppression or graft rejection). A large number of predisposing factors reflect the complex nature of risk observed in this group [133]. The enhanced risk of cardiovascular disease in patients with a transplanted kidney is associated with the accumulation of atherogenic risk factors before and after transplantation but also the use of immunosuppressive drugs. The presence of cardiovascular disease before the procedure is the predominant risk factor for the progression of this disease after transplantation. Before the transplantation, uremic milieu promotes the development of atherosclerosis and cardiovascular complications. In post-transplant patients, traditional cardiovascular risk factors are accompanied by risk factors linked with transplantation status and treatment (such as graft rejection, immunosuppressive agents, and cytomegalovirus infection) and those resulting from progressive regression in allograft function (oxidative stress, anemia, volume load, inflammation and proteinuria, hyperhomocysteinemia, secondary hyperparathyroidism) [133,134]. Patients with chronic kidney disease (CKD), and especially those undergoing dialysis exhibit increased cardiovascular (CV) risk compared to the general population [135,136]. Hemodialysis (HD) patients experience a 10–20 times higher risk of cardiovascular disease (CVD) mortality [137]. Transplantation has been shown to reduce cardiovascular risk compared to HD, however, this risk is still high [138]. The term “cardiovascular disease” covers coronary artery disease (CAD), congestive cardiac failure (CCF), peripheral vascular disease and cerebrovascular disease [136]. According to studies, the rate of cardiac death is estimated to be 10-times higher in renal transplant recipients compared with the general population, while the annual prevalence of fatal or nonfatal CV events is 50-times higher [139]. Cardiac disease is responsible for 17% of all deaths in renal transplant recipients, while cerebrovascular disease accounts for another 5% of all deaths. Cardiac arrest (45%) and then myocardial infarction (MI) (31%) and cardiac arrhythmia (13%) are the most frequent cardiac causes of death in this group of patients [140]. The incidence of myocardial infarction following transplantation is still high (6.5–11.1% at 36 months) and reaches its peak within the first 6 months from the procedure [141]. Aftab et al. [142] revealed that 86% of major adverse cardiac events occurred within the first 180 days. Moreover, it has been demonstrated that the development of post-transplant diabetes worsens graft function which in consequence leads to enhanced morbidity and mortality, especially from CV events [6]. Diabetes and PTDM partly contribute to the overall CV risk. Transplant recipients with post-transplant diabetes or impaired glucose tolerance have been shown to have an enhanced risk of developing CVD, but in those who suffered from diabetes pretransplantation, this risk is even greater [6,43]. In renal transplant, patients’ prediabetes is the main risk factor for PTDM, however, its impact on cardiovascular disease remains unclear [16,28,143]. The elusive mechanism of the relationship between prediabetes and cardiovascular disease may involve obesity, insulin resistance, hypertension, inflammation, and high triglyceride levels, all resulting in a pro-atherogenic state stimulating cardiovascular disease [144]. Porrini et al. [19] revealed that prediabetes (IFG and/or IGT) present at 12 months from transplantation posed an independent risk factor for cardiovascular events. In their study, the risk was two times higher in patients with prediabetes compared to those with normal glucose metabolism (HR 2.12, CI 1.14–3.93). The observed cardiovascular disease risk and also the survival curves for both complications were similar in prediabetes (HR 2.11, 95% CI 1.14–3.93) and PTDM (HR 2.24, 95% CI 1.11–4.52) [19]. Early glucose metabolism impairment, including altered fasting glucose observed within the first week after transplant, may also predict a higher risk of developing PTDM [145]. However, the early reversibility rate of IFG or IGT could be the result of the lack of association between prediabetes at 3 months with cardiovascular disease in some studies [19]. A systematic review and meta-analysis of 53 studies comprising 1,611,339 individuals indicated that prediabetes was related to a 15–30% enhanced risk for cardiovascular events [146]. In the study of 1410 consecutive transplant recipients who underwent repeated oral glucose tests (OGTTs) Valderhaug et al. [147] observed that IGT at 10 weeks after transplantation was associated with risk for total mortality but not with cardiovascular disease. However, Wauters et al. [115] found the correlation between impaired fasting glycemia (IFG) at 12 months and risk of major cardiovascular events (cardiac (hazard ratio (HR) = 1.113 (1.094–1.132), *p* < 0.0001), vascular (HR = 1.168 (1.140–1.197), *p* < 0.0001), and strokes (HR = 1.156 (1.123–1.191), *p* = 0.003), which was independent of other risk factors. Moreover, hyperglycemia also increased the risk of death (PTDM: HR = 2.410 (1.125–5.162), *p* = 0.024) in patients with major cardiovascular events after transplantation (*n* = 123, 11%). In another study, increasing fasting glucose levels at 1, 4, and/or 12 months from transplantation were significantly associated with CV events and these relationships were independent of other CV risk factors, including male gender, advanced age, CV events before transplantation and dyslipidemia [29]. Moreover, fasting glucose levels >100 mg/dL related to higher incidence of post-transplant cardiac (*p* = 0.001) and peripheral vascular disease events (*p* = 0.003).

**Figure 1:**
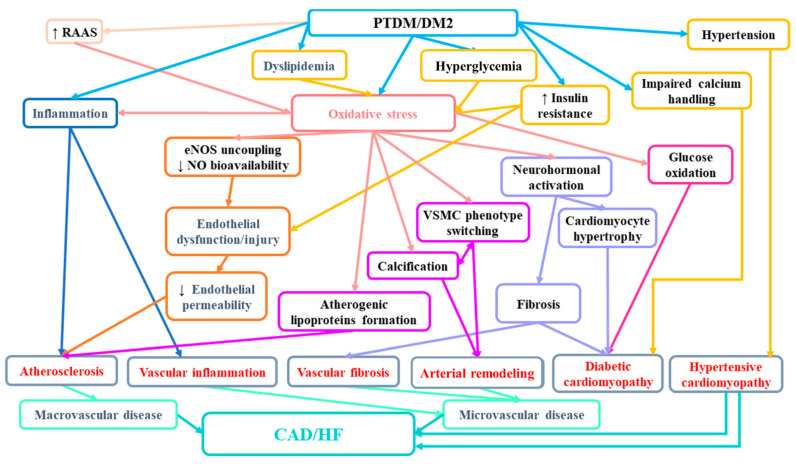
The suggested mechanisms involved in increased CAD/HF risk in patients with PTDM/diabetes mellitus. RAAS—renin-angiotensin-aldosterone system; eNOS—endothelial nitric synthase; NO—nitric oxide; VSMC—vascular smooth muscle cells; CAD—cardiovascular disease; HF—heart failure.

In the light of the evidence, it seems that not only PTDM but also prediabetes may play an important role in the burden of cardiovascular disease in the population of renal transplant patients. Therefore, the diagnosis of prediabetes with a simple tool, such as the OGTT, may enable the identification of patients at risk and help to avoid cardiovascular complications in this population. Figure 1 presents mechanisms involved in enhanced cardiovascular risk in patients with diabetes.

## 6. The Assessment of PTDM Risk and Recommendations Concerning Its Diagnosis and Management

The assessment of the risk of PTDM should preferably be performed before kidney transplantation to enable early implementation of the appropriate intervention [97]. The results of studies indicated that the use San Antonio Diabetes Prediction Model and the Framingham Offspring Study-Diabetes Mellitus algorithm quite effectively identified patients at higher risk for PTDM beyond the first year [148]. In 2014, the International Expert Panel suggested that screening tests for PTDM should involve also postprandial glucose monitoring and HbA1C. However, HbA1C test should not be used early after transplantation (within 45 days from transplantation) due to potential confounding factors [16]. Normal value of HbA1C test does not exclude the diagnosis of PTDM in the presence of early post-transplant anemia and/or dynamic kidney allograft function [25]. In the opinion of Alnasrallah et al. [1] performing OGTTs after renal transplantation is recommended even in nondiabetic patients. According to other studies, homeostasis model assessment of insulin resistance and OGTT does not adequately reflect the altered carbohydrate metabolism of individuals with chronic kidney disease (CKD) or ESRD since in these patients, endogenous insulin concentrations may be increased due to impaired renal clearance of endogenous insulin [97].

The reduction of type 2 diabetes mellitus is related to the introduction of lifestyle interventions that promote decreased fat/energy consumption, moderate-intensity physical activity, and moderate weight loss [97]. According to two large studies, lifestyle intervention reduced the incidence of diabetes by 58% compared with placebo [149,150]. However, it is not obvious whether the aforementioned modification of diet and introducing physical activity can also prevent or delay the progression of PTDM. The results of some studies imply that intensive lifestyle modifications that are tailored to the requirements of patients with CKD or ESRD and which are delivered before and straightaway after transplantation might decrease the incidence of PTDM [97,151]. Due to the fact that higher BMI before the transplantation correlates with the development of insulin resistance after transplantation, it seems that obesity should be treated as the target for intervention to prevent PTDM. Current guidelines concerning the management of prediabetes or diabetes recommend lifestyle modifications enabling overweight or obese patients to lose weight [152]. Due to the fact that the maintenance of proper weight after its loss (arising from intensive lifestyle interventions) is difficult, guidelines suggest (in certain cases) the administration of pharmacological antidiabetic therapy immediately after the diagnosis of diabetes [152,153]. For example, Alnasrallah et al. [1] suggest introducing metformin therapy in transplant patients with IGT. Metformin has been demonstrated to exert favorable effects in this group of patients since it not only improves insulin sensitivity but also influences the cardiovascular system and weight gain [154,155]. In turn, Guthoff et al. [20] suggested that overweight patients with insulin resistance might benefit from steroid-free maintenance immunosuppression, due to the fact that corticosteroids negatively influence insulin sensitivity. They claim that the use of corticosteroid-free maintenance immunosuppression for standard immunological risk patients give outstanding long-term allograft survival. Due to the fact that calcineurin inhibitors directly impair β-cell function, a low-dose CNI or CNI-free regimen seems to be beneficial in patients with impaired insulin secretion and normal immunological risk.

After solid-organ transplantation, especially in the immediate post-transplant period, the incidence of “stress hyperglycemia” is high, however, there are no sound recommendations concerning the management strategies for post-transplant hyperglycemia and subsequent outcomes [156,157]. The rationale for hyperglycemia management is to limit stress on β-cells during the peri-transplant period in order to expand their long-term function [158]. Since compromised insulin secretion seems to be the major pathophysiological feature after kidney transplant, early therapeutic interventions aiming to preserve, maintain, or enhance β-cell function should be potentially undertaken in this population [15]. Several preventive strategies have been suggested to improve insulin secretion via the protection and amelioration of βcell function. βcell function seems to be protected when basal insulin is administered perioperatively. Hecking et al. [158] demonstrated that the treatment group showed significantly improved βcell function (assessed on the basis of insulinogenic index) but not insulin sensitivity, even 1 year after transplantation, despite the fact that insulin administration was ceased 3 months after the transplantation. The results of the randomized controlled trial of 50 renal transplant recipients revealed that early (<3 weeks) administration of basal insulin to treat post-transplant hyperglycemia considerably diminished the risk of developing PTDM within the first year by 73% [158]. However, there is no consensus regarding long-term glycemic targets for heart transplant recipients with PTDM [24].

Other studies recommended the use of incretins which have been found to exert a protective effect on βcells and improve clinical outcome in pancreatic islet cell transplantation, however, the efficacy and safety of incretin analogues and dipeptidyl peptidase (DPP)-4 inhibitors have not yet been established in solid organ transplantation [159,160,161].

Table 2 presents the results of studies described in this paragraph.

According to the prepared but not yet accepted Guidelines On The Detection And Management Of Diabetes Post Solid Organ Transplantation Detection of Association of British Clinical Diabetologist and Renal Association, the diagnosis of PTDM should be avoided in the immediate post-operative period when transient hyperglycemia is extremely common (Grade 1B) and thus a formal diagnosis can be made from 6-weeks post-transplantation (Grade 1B) [114]. The identification of patients with post-operative hyperglycemia should be made with the use of afternoon capillary blood glucose monitoring (AGM) and such patients ought to be closely monitored for PTDM (Grade 1B). Currently, oral glucose tolerance test is the gold standard for the diagnosis of PTDM, and HbA1c ≥6.5% (48 mmol/L) is a proper diagnostic test in clinically stable solid organ transplant recipients after the first 3 months post-transplantation (grade 1B). However, the results of the latter test should be used with caution since some factors may impair its accurate interpretation (Grade 1A). Results of abnormal fasting plasma glucose (FPG) ≥7 mmol/L and/or HbA1c >6.5% (48 mmol/mol) allows for the identification of the majority of PTDM cases in stable patients (Grade 2C). Patients awaiting transplant ought to receive annual glycemic testing with FPG ± HbA1c. Those at high risk patients should then undergo OGTT to confirm diagnosis of diabetes or to screen for impaired glucose tolerance (Grade 2C). The new guidelines do not recommend the use of novel diagnostic tools (e.g., fructosamine and glycated albumin) as clinical tools (Grade 2D). After transplantation, early postoperative hyperglycemia (glucose >11 mmol/L on two occasions within 24 h) should be straightaway actively monitored and treated with oral hyperglycemic therapy if it is mild (<14.0 mmol/L), or with early intravenous or subcutaneous insulin therapy in more severe cases (Grade 1C). Glycemic target for patients with PTDM should be established at c.a. 7% (53 mmol/mol), however, while setting the target the severity of CKD, comorbidities, age and the ability to self-manage ought to be taken into consideration (Grade 1B). Patients with a confirmed diagnosis of PTDM should be offered structured diabetes education as well as structured diabetes care, involving regular screening for complications (Grade 1B). Additionally, blood pressure should be controlled below 130/80 mmHg in all patients with PTDM (Grade 1B) [114].

Metformin should be considered first-line oral therapy for patients with confirmed PTDM and a stable eGFR > 30 mL/min/1.73 m^2^ and BMI > 25 kg/m^2^ (Grade 1C). Sulfonylureas, meglitinides, DPP-4 inhibitors, pioglitazone, and GLP-1 analogue can be safely used in patients with PTDM, however, caution should be exercised while using the first two drugs in those at risk of hypoglycemia (Grade 2C). In patients with stable eGFR and poor glycemic control, SGLT-2 inhibitors should be used with caution, after consultation with a nephrologist and a diabetologist (Grade 1C). In turn, insulin therapy should be considered in all patients with insufficient glucose control, or with symptomatic hyperglycemia (Grade 1C). Irrespective of cholesterol level, all patients suffering from PTDM ought to be provided with statin therapy (Grade 2D) [114].

Since immunosuppression is the main risk factor for PTDM, its modifications can be made to reduce this risk, however, the advantages must be balanced against the risk for allograft rejection (Grade 1B). The strategy to improve long-term transplant outcomes should involve the adjustment of immunosuppression on the basis of the recipient’s immunologic and glycemic risk (Grade 1C). For the time being, due to a lack of contradictory evidence, the selection of immunosuppressive therapy should be principally targeted at the prevention of rejection rather than preventing PTDM (Grade 1C) [114]. Finally, in patients before the transplantation, the risk for diabetes development should be assessed (Grade 1B) and such patients ought to be educated on the risk of developing PTDM as well as counseled about minimizing weight gain using lifestyle measures (Grade 1B). In patients awaiting the transplantation, the treatment of PTDM risk factors, including hepatitis C should be introduced (Grade 1C). Moreover, all patients who are at high risk for the development of PTDM should be screened yearly for diabetes whilst awaiting transplantation (Grade 1B) [114].

**Figure 2:**
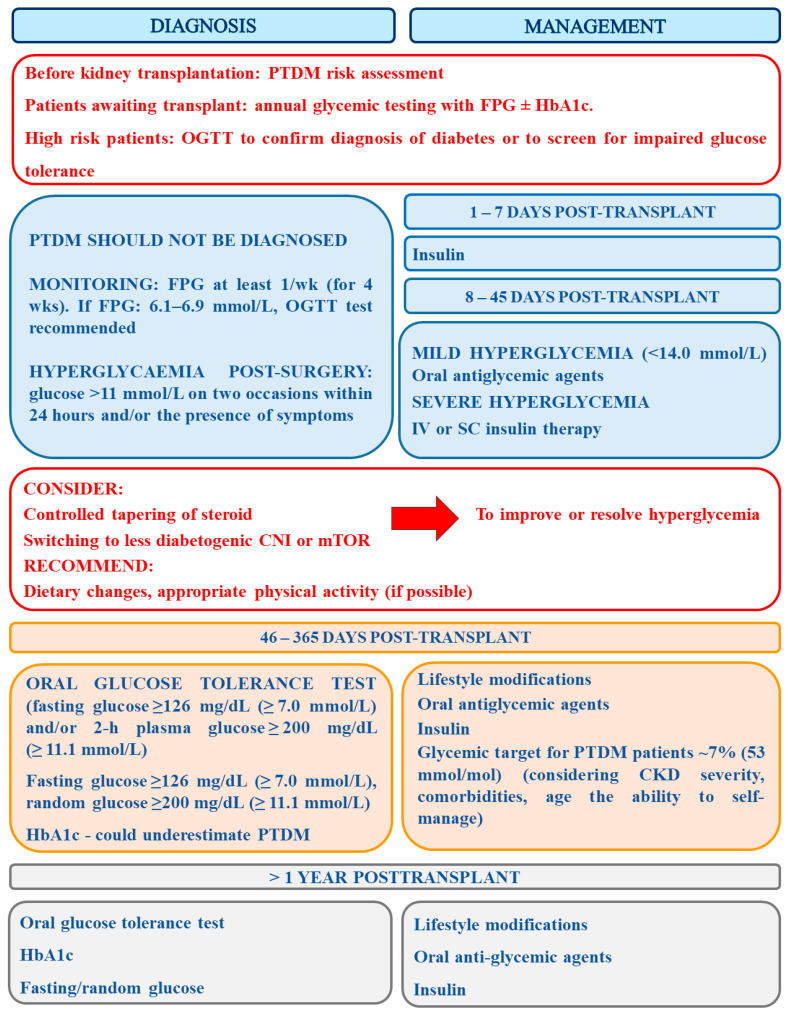
The summary of recommendations concerning PTDM diagnosis and management (prepared on the basis of [41,112,114].

The summary of recommendations concerning PTDM diagnosis and management is presented in Figure 2.

Recently, the use of SGLT inhibitors has attracted great interest. The sodium-glucose cotransporters (SGLTs), located on the apical membrane of renal proximal tubule cells, participate in tubular reabsorption of the filtered glucose [163,164]. There are two types of these cotransporters—SGLT2 displaying low affinity but high capacity for glucose and reabsorbing ≈80–90% of the filtered glucose load under normal conditions and SGLT1 which has a high affinity but low capacity for glucose, and it is responsible for the reabsorption of the remaining 10–20% of the filtered glucose [165,166]. In poorly controlled diabetic subjects, the filtered glucose load can exceed maximum reabsorptive capacity for glucose, resulting in glucosuria. Greater reabsorption of glucose worsens hyperglycemia in patients with T2DM [162,167]. Recently, it has been suggested that the inhibition of renal glucose reabsorption is an efficient method of improving glycemic control, βcell function and insulin sensitivity in patients with type 2 diabetes mellitus (T2D) [163,168,169]. The results of studies of animal models of T2DM demonstrated enhanced mRNA expression of SGLT1 and SGLT2 in the kidney, its correlation with glycemia and HbA1c and attenuation following the administration of hypoglycemic agents [170,171]. Norton et al. [163] suggested that therapies hindering both SGLT1 and SGLT2 could be more efficient in the improving of glycemic control in patients with T2DM than it was previously hypothesized [172].

Sotagliflozin, which is a dual sodium-glucose cotransporter (SGLT)1/SGLT2 inhibitor, has been recently approved in Europe as an adjunct to insulin therapy in adults with type 1 diabetes (T1D) and a body mass index (BMI) ≥ 27 kg/m^2^ [173]. According to studies, concomitant use of 200 and 400 mg of Sotagliflozin and insulin decreased glycated hemoglobin level and lowered body weight and systolic blood pressure. The administration of this drug is associated with diminished occurrence of severe hypoglycemia and documented hypoglycemia ≤3.1 mmol/L events, but also with elevated incidence of diabetic ketoacidosis in patients with BMI ≥ 27 kg/m^2^. Additionally, in the phase 3, in TANDEM 1–3 trials, adjunctive use of oral sotagliflozin was associated with beneficial effects (better glycemic control, body mass reduction) which were maintained over 52 weeks of treatment. This trial demonstrated that this inhibitor was well tolerated and diminished the likelihood of hypoglycemia [174]. On the basis of its risk/benefit profile, sotagliflozin is indicated in the EU as an adjunct to insulin in adults with T1D with a BMI ≥ 27 kg/m^2^ who have failed to achieve adequate glycemic control despite optimal insulin therapy, thus expanding the currently limited adjunctive oral treatment options available for use in this population. The results of meta-analysis of 13 studies (7962 participants) involving the use of SGLT inhibitors demonstrated that they facilitate glycemic control with a decreased insulin dose [175]. Moreover, in comparison with a placebo, the effects of the use of a dual SGLT inhibitor (sotagliflozin) on type 1 diabetes are similar to those of SGLT2 inhibitors, however, this type of inhibitor did not rise the risk of genital infections. The meta-analysis indicated that SGLT inhibitor treatment enhanced the incidence of urinary tract and genital infections, diarrhea, and diabetic ketoacidosis [175]. The inhibition of SGLT1 is associated with the blockage of glucose in the intestine, followed by its breakage down into short-chain fatty acids by bacteria at the distal end of the small intestine, which stimulate the secretion and release of glucagon-like peptide-1 and peptide YY by L cells at the distal end of the intestine [176]. Due to the fact that SGLT inhibitors promote the excretion of a large amount of glucose through the urine rising glucose concentration in the genitourinary tract, they enhance the risk of bacterial and fungal infection in patients [175]. In turn, the risk of diabetic ketoacidosis observed in patients using SGLT inhibitors is probably associated with their impact on the secretion of insulin and glucagon, stimulation of the decomposition of adipose tissue and β-oxidation of fatty acids and consequent enhanced formation of ketones in the liver [177]. Moreover, these inhibitors promote the lipid mobilization and free fatty acid oxidation in vivo, raise the level of free fatty acid and 13-hydroxybutyric acid in plasma, diminish the removal of ketone bodies via the kidneys, and enhance the reabsorption of ketone bodies in the proximal convoluted tubules [175,178]. Therefore, such therapy should be used with caution in patients who suffer from recurrent urogenital infections, ketosis or acidosis [175].

Based on the risk/benefit profile, sotagliflozin is approved in the EU as an adjunct to insulin in adult patients with T1D with a BMI ≥ 27 kg/m^2^ who failed to accomplish adequate glycemic control despite optimal insulin therapy [174]. According to the present ABCD/Diabetes UK joint updated position statement, clinicians should help patients with type 1 diabetes using these drugs to mitigate this risk and other potential complications [179]. Especially patients who are at risk of diabetic ketoacidosis due to illnesses, low calorie diets, starvation, injuries, excessive exercise, reduced insulin administration, excessive alcohol consumption, and other factors increasing the risk for diabetic ketoacidosis should be treated with utmost caution.

To sum up, sotagliflozin therapy as an adjunct to optimized insulin treatment in overweight/obese patients with T1D was shown to address some unmet needs and to enable the achievement of optimal glycemic control [173]. However, there are no available publications concerning the use of these inhibitors in patients with PTDM.

## 7. Conclusions

Kidney transplantation seems to be the best therapy for ESRD, however, it is not deprived of drawbacks, including the development of PTDM which affects allograft and patient survival [97]. Management of post-transplant diabetes resembles that of diabetes in the general population as it is based on strict glycemic control as well as screening and treatment of common complications [136]. Lifestyle intervention accompanied by the tailoring of immunosuppressive regimen may be of key importance to mitigate PTDM-associated complications in kidney transplant patients [33,180,181]. A more transplant-specific approach can include the change of tacrolimus by an alternative immunosuppressant (cyclosporine or mTOR inhibitor), the decrease or cessation of corticosteroid therapy [69], and caution in the prescribing of diuretics since they are independently connected with post-transplant diabetes [182,183]. Moreover, early administration of basal insulin has been shown to considerably decrease the risk of PTDM, which might be related to insulin-mediated β-cell protection and “resting” [158].

Early identification of high-risk patients for cardiovascular diseases enables a timely introduction of appropriate therapeutic strategy and results in higher survival rates for patients with a transplanted kidney.

A lot is known about PTDM, however, still numerous topics require clarification. First of all, future large studies are necessary to reveal all risk factors associated with the development of PTDM. The good knowledge of genetic alterations that increase the risk of PTDM may enable, in the future, the development of personalized therapy of PTDM. The differences in risk factors between genders should be confirmed in large multicenter studies. It also seems interesting whether recipients of gender-mismatched organs are at higher risk of the development of PTDM. Moreover, the long-term outcomes of transplant patients with PTDM across different populations also need research. Do patients with PTDM suffer from the same complications and to the same extent as patients with standard type 2 diabetes mellitus? Additional prospective studies are necessary to understand the associations between immunosuppressant regimens and PTDM and to develop the strategy considerably lowering the hazard of PTDM. Better characterization of clinical grounds of PTDM will enable its early detection and the introduction of appropriate treatment as well as the prevention of devastating complications.

Finally, emerging evidence has indicated the relationship between the exposure of endocrine-disrupting chemicals (EDCs) and diabetes [184]. EDCs were suggested to play a vital role in the etiology of diabetes and metabolic disorders since these chemicals may disturb pancreatic endocrine system and glucose metabolism. Several epidemiological studies have revealed significant impact of EDCs and hyperglycemia, glucose intolerance, and insulin resistance [185,186,187]. The suggested course of action includes the interactions with the aryl hydrocarbon receptor (AhR) and nuclear hormone receptors (e.g., estrogen receptors), the alteration of ERK/Akt signaling pathways, the stimulation of oxidative and nitrosative stress, as well as pancreatitis and dysregulated hepatic metabolism [188]. Additionally, changes in the gut microbiota have been shown to contribute to the onset and maintenance of insulin resistance [189]. Alterations in intestinal ecosystem could stimulate inflammation, modify intestinal permeability, and modulate metabolism of bile acids, short-chain fatty acids and metabolites which synergistically exert impact on metabolic regulation systems thus promoting insulin resistance [189]. It has been suggested that interventions which restore the equilibrium in the gut may have beneficial effects and improve glycemic control. However, future research should be performed to reveal identify exact pathophysiological mechanisms of EDC and gut dysbiosis, discover new potential therapeutic targets and assess their impact on the strategies that reduce dysbiosis and improve glycemic control.

## Figures and Tables

**Table 1:** The summary of post-transplant diabetes mellitus (PTDM) risk factors and involved pathomechanisms described in this manuscript.

Type of Study	Study Group	Most Important Results	S—Strong Evidence, A—Association	References
Risk Factors				
Prospective cohort study	A total of 487 RTR (age 50 ± 12 years, 55% men); 16% developed PTDM	⮚ Proinsulin (hazard ratio, 2.29; 95% CI, 1.85–2.83; *p* < 0.001) strongly associated with NODAT development independently of age, sex, calcineurine inhibitors, prednisolone use, components of the metabolic syndrome, or homeostasis model assessment. Conclusions: Proinsulin (a marker of β-cell dysfunction) is strongly related to development of NODAT in RTR. Confirmation of the role of β-cell dysfunction in the pathophysiology of NODAT in RTR.	S	[32]
Single-center cohort study	450 recipients of living and deceased donor kidney transplants on immunosuppressive therapy; 13.5% developed PTDM	⮚ Pretransplant variables associated with PTDM: recipient age (46:2 ± 1:3 vs. 40:7 ± 0:6 years old, OR 1.04; *p* = 0.001) and pretransplant hyperglycemia and BMI ≥ 25 kg/m^2^ (32.8% vs. 21.6%, OR 0.54; *p* = 0.032 and 57.4% vs. 27.7%, OR 3.5; *p* < 0.0001, respectively). ⮚ Posttransplant transient hyperglycemia (86.8%. 18.5%, OR 0.03; *p* = 0.0001), acute rejection (*p* = 0.021), calcium channel blockers (*p* = 0.014), TG/HDL ratio ≥ 3:5 at 1 year (*p* = 0.01) and at 3 years (*p* = 0.0001), and tacrolimus trough levels at months 1, 3, and 6 were equally predictors of PTDM. ⮚ Multivariate analyses: pretransplant hyperglycemia (*p* = 0.035), pretransplant BMI ≥ 25 kg/m^2^ (*p* = 0.0001), post-transplant transient hyperglycemia (*p* = 0.0001), and TG/HDL ratio ≥ 3:5 at 3-year post-transplant (*p* = 0.003) were associated with PTDM diagnosis and maintenance over time. Conclusions: Early identification of pretransplant hyperglycemia and overweight, post-transplant transient hyperglycemia, tacrolimus trough levels, and TG/HDL ratio may be useful for risk stratification of patients to determine appropriate strategies to reduce PTDM.	A	[33]
Observational study	359 de novo renal allograft recipients; 17.8% developed PTDM (follow-up 42.8 ± 16.9 months)	⮚ Independent risk factors for PTDM: age (OR: 1.05 (95% Cl: 1.019–1.083)), BMI (OR: 1.09 (1.013–1.189)), proteinuria on Day 5 (OR: 1.51 (1.043–2.210)) and BPAR (OR: 2.74 (1.345–5.604)). ⮚ Factors related with lower PTDM risk: normal OGTT on Day 5 post-transplantation (OR: 0.03 (0.008–0.166)) and normal FPG on Day 5 (OR: 0.06 (0.012–0.338)). Conclusions: The Day 5 OGTT can be used for identifying recipients at reduced risk for PTDM, taking into account the impact of independent clinical risk factors like age, BMI and BPAR treatment.	A	[34]
Systematic review of the published medical literature of the relationship between anti-HCV seropositive status and DM after RT	2502 unique RT recipients were identified. The incidence of PTDM after RT ranged between 7.9% and 50%	⮚ Significant link between anti-HCV seropositive status and DM after RT—one potential explanation for the adverse effects of HCV on patient and graft survival after RT. Conclusions: HCV can be included in the subset of potentially modifiable risk factors for PTDM after RT.	S	[38]
In vitro study	Virus infection system/insulinoma cell line, MIN6	⮚ HCV virion has a dose- and time-dependent cytopathic effect on the cells. ⮚ HCV infection inhibits cell proliferation and induces death of MIN6 cells. ⮚ HCV infection also causes endoplasmic reticulum (ER) stress. ⮚ HCV RNA replication was detected in MIN6 cells, although the infection efficiency is very low and no progeny virus particle generates. Conclusions: HCV infection induces death of pancreatic beta cells through an ER stress-involved, caspase 3-dependent, special pathway.	S	[39]
Observational study	386 adult kidney transplant recipients from Canadian kidney transplant population; cumulative incidence rate of PTDM—9.8%	⮚ Five clinical factors were independently associated with PTDM: older recipient age, deceased donor, hepatitis C antibody status, rejection episode and use of tacrolimus (vs. cyclosporine).	S	[41]
Case–control study	2078 non-DM renal allograft recipients; 21% developed PTDM	⮚ More rapid increase in PTDM correlated with: older age (RR = 2.2 comparing patients younger or older than 45 years, *p* < 0.0001), transplant done after 1995 (RR = 1.7, *p* = 0.003), African American race (RR = 1.6, *p* = 0.003), and higher body weight at transplant (RR = 1.4, *p* < 0.0001).	S	[43]
Retrospective study	Group 1, SIR plus full-exposure CSA/S (*n* = 118); group 2, full-exposure CSA/S/no SIR ± antiproliferative drug (*n* = 141); group 3, SIR plus reduced CSA exposure/S (*n* = 212); group 4, no SIR/full-exposure CSA/S ± antiproliferative drug (*n* = 43)	⮚ NODAT rates reflected the level of CSA exposure; at 10 years 54% versus 30% for groups 1 versus 2 (*p* = 0.0001); at 5 years 30% versus 21% for Groups 3 versus 4 (*p* = 0.3). ⮚ Reduced CSA exposure had beneficial effects (*p* = 0.02; HR, 1.006). ⮚ Differences in steroid treatment did not play a significant role in NODAT. ⮚ SIR was an independent risk factor for NODAT (*p* = 0.004; HR, 3.5). Conclusions: SRL is an etiologic agent for NODAT, displaying interactive, possibly pharmacokinetic, and pharmacodynamic effects with concomitant CsA in combination treatment.	S	[62]
Prospective study	173 consecutive kidney transplant recipients	⮚ High incidence of PTDM (18%) and IGT (31%). ⮚ Age, family history of diabetes, HLA-B27 phenotype, DR mismatch, rejection, actual prednisolone dose, total methylprednisolone dose, total steroid dose, cytomegalovirus (CMV) infection, and the use of furosemide were associated with PTDM. ⮚ The risk of developing PTDM was 5% per 0.01 mg/kg/day of increase in prednisolone dose. Conclusions: Increased prednisolone dose and older age are strongly associated with the development of posttransplant glucose intolerance.	S	[64]
Retrospective study	11,659 Medicare beneficiaries from the United Renal Data System who received their first kidney transplant	⮚ Risk factors for PTDM included age, African American race (relative risk = 1.68, range: 1.52–1.85, *p* < 0.0001), Hispanic ethnicity (1.35, range: 1.19–1.54, *p* < 0.0001), male donor (1.12, range: 1.03–1.21, *p* = 0.0090), increasing HLA mismatches, hepatitis C infection (1.33, range: 1.15–1.55, *p* < 0.0001), body mass index > or = 30 kg/m^2^ (1.73, range: 1.57–1.90, *p* < 0.0001), and the use of tacrolimus as the initial maintenance immunosuppressive medication (1.53, range: 1.29–1.81, *p* < 0.0001). Conclusions: High incidences of PTDM are associated with the type of initial maintenance immunosuppression, race, ethnicity, obesity, and hepatitis C infection.	S	[66]
Retrospective study	177 adult patients, without previously known diabetes who underwent transplantation	⮚ Variables associated with the onset of PTDM: older recipient age (*p* = 0.05), male gender (*p* = 0.03), family history of diabetes (=0.04), advanced donor age (*p* = 0.008), absence of induction immunosuppression (*p* = 0.04), use of tacrolimus (vs. cyclosporine; *p* = 0.01), one or more than one (steroid-treated) acute rejection episode(s) (*p* = 0.000001), cytomegalovirus infection (*p* = 0.02), and use of β-blockers or diuretics (*p* = 0.05). Conclusions: Apart from traditional risk factors predisposing to the development of type 2 diabetes in the general population, also episodes of acute rejection significantly influence the incidence of PTDM.	S	[70]
Case–control study	315 renal transplant recipients	⮚ Significant association between 223Arg variant and PTDM risk (OR = 3.26 (1.35–7.85), *p* = 0.009) after correcting for multiple testing. ⮚ BMI at transplant was associated with PTDM (*p* > 0.00001). Conclusions: Genetic variability in the LEPR may contribute significantly to the risk for PTDM in renal transplant recipients. The effect of the 223Arg variant on PTDM is strongly modulated by the age of the recipient.	A	[71]
Case–control study	129 nondiabetic, primary, Chinese Han renal allograft recipients treated with TAC; 13.2% developed PTDM	⮚ Age over 50 years old and CYP24A1 rs2296241 A allele were independently correlated with the development of PTDM. Conclusions: Patients with advanced age and CYP24A1 rs2296241 A allele had an increased risk of PTDM after kidney transplantation.	A	[72]
Case–control study	Hispanic kidney allograft recipients without evidence of preexisting diabetes who developed NODAT	⮚ Hepatocyte nuclear factor 4 alpha (HNF4A) AA (rs2144908, OR 1.96, CI 1.08–3.50, *p* = 0.010), HNF4A TT (rs1884614, OR = 2.44, CI 1.42–4.48, *p* = 0.002), and insulin receptor substrate 1 AA + AG (rs1801278, OR 2.71, CI 1.16–6.89, *p* = 0.021) remained significant after logistic regression. Among the clinical factors, average age (OR 1.01, CI 1.00–1.08, *p* = 0.048), sirolimus (OR 5.36, CI 3.02–10.4, *p* = 0.001), deceased donor (OR 1.96, CI 1.16–2.94, *p* = 0.015), and acute rejection (OR 2.92, CI 1.31–5.77, *p* = 0.009) remained significant after logistic regression. Conclusions: Polymorphism of two alleles of HNF-4A gene and insulin receptor substrate 1 are significantly associated with PTDM in kidney transplant patients with Hispanic ethnicity.	A	[73]
Case–control study	323 patients who received kidney transplants and treated with tacrolimus or cyclosporine	⮚ LEP rs2167270 gene polymorphism was statistically significantly associated with increased risk of PTDM. Conclusions: Alterations in leptin gene may affect the risk of PTDM.	A	[74]
Comparative study	168 nondiabetic patients (58% males, 69% of Chinese ethnicity) who received renal transplantation	⮚ Increased risk of PTDM in renal-transplant patients receiving higher daily dose of cyclosporine (HR = 1.01 per mg increase in dose, 95% CI 1.00–1.01, *p* = 0.002). ⮚ Gender, ethnicities, age at transplant, primary kidney disease, type of donor, place of transplant, type of calcineurin inhibitors, duration of dialysis pretransplant, BMI, creatinine levels, and daily doses of tacrolimus and prednisolone were not significantly associated with risk of PTDM. ⮚ GA genotype of rs1494558 (HR = 3.15 95% CI 1.26, 7.86) and AG genotype of rs2232365 (HR = 2.57 95% CI 1.07, 6.18) were associated with increased risk of PTDM as compared to AA genotypes. Conclusions: The daily dose of cyclosporine and SNPs of IL-7R (rs1494558) and MBL2 (rs2232365) genes are significantly associated with the development of PTDM in the Malaysian renal transplant population.	A	[77]
Comparative study	306 renal transplants recipients without a history of diabetes	⮚ Alleles: rs2069763*T (IL-2), rs1494558*A and rs2172749*C (IL-7R), and rs4819554*A (IL-17R) were significantly higher in patients with PTDM. ⮚ 11 SNPs (IL-1B (rs3136558), IL-2 (rs2069762), IL-4 (rs2243250, rs2070874), IL-7R (rs1494558, rs2172749), IL-17RE (rs1124053), IL-17R (rs2229151, rs4819554), and IL-17RB (rs1043261, rs1025689) were significantly associated with PTDM development after adjusting for age, sex, and tacrolimus usage. Conclusions: Inflammation of islet β cells might play a crucial role in the pathogenesis of PTDM in renal transplantation recipients. Significant variations of IL-7R, IL-17E, IL-17R, and IL-17RB could be associated with the pathogenesis of PTDM in renal transplant recipients.	A	[80]
Comparative study	278 renal transplant participants, including 251 subjects free of diabetes and 27 with PTDM	⮚ Patients with the IL-6 G/G genotype experienced a lower risk of developing PTDM (OR 0.08; 95% CI 0.01–0.86). Conclusions: The G/G genotype of IL-6 may play an important role to lower the risk for PTDM development.	A	[81]
Comprehensive meta-analysis of data from 36 publications	Kidney transplant recipients	⮚ Polymorphisms significantly associated with PTDM at the 5% level of significance: CDKAL1 rs10946398 *p* = 0.006 OR = 1.43, 95% CI 1.11–1.85 (*n* = 696 individuals), KCNQ1 rs2237892 *p* = 0.007 OR = 1.43, 95% CI 1.10–1.86 (*n* = 1270 individuals), and TCF7L2 rs7903146 *p* = 0.01 OR = 1.41, 95% CI 1.07–1.85 (*n* = 2967 individuals). Conclusions: CDKAL1 (rs10946398), KCNQ1 (rs2237892) and TCF7L2 (rs7903146) are significantly associated with PTDM.	A	[82]
Comparative study	Hispanic renal transplant patients	⮚ T allele associated with tagging SNP for one of the five dominant NFATc4 haplotypes, T-T-T-T-G (rs10141896) was associated with a lower cumulative incidence of PTDM (*p* = 0.02). ⮚ CNI-treated recipients with this haplotype had a reduced adjusted risk for PTDM (HR: 0.45; 95% Cl: 0.19–1.01). ⮚ Patients homozygous for the C-C-C-G-G haplotype were at an increased risk (HR: 2.13; 95% Cl: 1.01–4.46) for PTDM in subanalysis. ⮚ The use of tacrolimus, sirolimus, and older age were associated with increased risk for PTDM. Conclusions: Polymorphisms in the NFATc4 gene may confer certain protection or predisposition for PTDM.	A	[88]
Comparative study	315 patients who received kidney transplants treated with calcineurin inhibitors, with PTDM (*n* = 43) and without PTDM (*n* = 272)	⮚ Significant positive association between hazard of PTDM development and the number of CCL2 rs1024611 G alleles (HR 1.65; 95%CI 1.08–2.53; *p* = 0.021). ⮚ This polymorphism is an independent risk factor for post-transplant diabetes. Conclusions: The results of our study suggest an association between the CCL2 gene rs1024611 G allele and PTDM in patients treated with tacrolimus or cyclosporine.	A	[89]
Comparative study	311 patients who had received kidney transplants without a prior history of diabetes; 18% developed PTDM	⮚ SNPs: rs2107538, rs2280789 and rs3817655 of the CCL5 gene were significantly associated with the development of PTDM in the codominant 2 and recessive models. ⮚ The frequency of the TCA haplotype was significantly higher in patients with PTDM than in those without PTDM. Conclusions: Genetic polymorphisms of the CCL5 gene are associated with PTDM, suggesting that the CCL5 gene might confer susceptibility to PTDM in patients who receive renal transplants.	A	[90]
Comparative study	302 subjects without previously diagnosed diabetes who had received kidney transplants; PTDM developed in 16.2%	⮚ rs4762 of the AGT gene was significantly associated with the development of PTDM in the dominant models (*p* = 0.03) after adjusting for age and tacrolimus usage. Conclusions: AGT gene rs4762 polymorphisms may serve as genetic markers for the development of PTDM, however, the exact molecular mechanisms still need to be clarified.	A	[91]
Comparative study	159 patients receiving kidney transplants, 21 developed PTDM	⮚ Allele T (SNP C599T (Pro200Leu)) in the GPX1 gene was significantly more frequent among patients with PTDM compared to patients without PTDM (OR = 2.14, 95% CI 1.11–4.12, *p* = 0.024). ⮚ No associations between SOD1, SOD2 and CAT polymorphisms and PTDM. Conclusions: Pro200Leu polymorphism of the GPX1 gene may be associated with the risk of PTDM development in renal graft recipients.	A	[92]
Comparative study	101 renal transplant recipients receiving tacrolimus-based immunosuppressive therapy	⮚ PPARα rs4253728A>G and POR*28 variant alleles were both independently associated with an increased risk for NODAT with respective odds ratios of 8.6 (95% CI = 1.4–54.2; *p* = 0.02) and 8.1 (95% CI 1.1–58.3; *p* = 0.04). Conclusions: Polymorphisms in PPARα and POR might predispose patients being treated with tacrolimus to the development of NODAT after kidney transplantation.	A	[95]
Retrospective study	218 records of postrenal transplant patients who had a minimum follow-up for 5 years. Patients with diabetes mellitus (DM; *n* = 21), PTDM (*n* = 58)	⮚ Risk factors of PTDM: recipient age >36 years, hepatitis C virus infection, HLA-B13, family history of DM, body mass index >30, and calcineurin inhibitor therapy. ⮚ PTDM group had reduced graft function compared with non-DM-non-PTDM subjects, when used glomerular filtration rate as marker. Conclusions: Regular screening of plasma glucose is recommended from the early transplant period, particularly among high-risk patients. Regular monitoring of graft function is necessary as PTDM influences graft function.	S	[101]
Comparative study	3365 adult kidney allograft recipients, group I (DM; *n* = 156), Group II (PTDM; *n* = 251) and Group III (nondiabetic; *n* = 2958)	⮚ Risk factors for developing PTDM: recipient age >60 years (OR = 2.24; *p* < 0.001), female recipient (OR = 1.5; *p* < 0.005), recipient weight >65 kg (OR = 1.77; *p* < 0.002), BMI > 25 kg/m^2^ (OR = 1.6; *p* < 0.04) or >30 kg/m^2^ (OR = 2.92; *p* < 0.005), and tacrolimus-based therapy (OR = 1.48; *p* < 0.05). The use of Sandimmune vs. Neoral had a protective effect (OR = 0.7; *p* < 0.01). ⮚ PTDM correlated with reduced patient survival (RR = 1.55; 95% CI = 1.05–2.27; *p* < 0.02). Conclusions: PTDM was an independent negative predictor of patient survival after kidney transplantation.	S	[102]
Comparative study	314 nondiabetic adults who received a renal allograft; PTDM developed in 16%	⮚ Prednisone dose (*p* = 0.0001, HR 1.007 (1.003–1.010) per 1 mg/d at 3 months), weight at transplant (*p* = 0.02, HR 1.022 (1.003–1.042) per 1 kg), black ethnicity (*p* = 0.02, HR 1.230 (1.023–1.480)) and age > or = 45 years (*p* = 0.01, HR 2.226 (1.162–4.261)) increased diabetes risk. ⮚ Statin use is associated with reduced new-onset diabetes development after renal transplantation.	A	[103]
Single-center retrospective study	633 nondiabetic patients receiving a first kidney transplant; 26.2% of recipients developed PTDM	⮚ Significantly higher FPG (*p* = 0.026) and BMI (*p* = 0.006) and lower HDL levels (*p* = 0.015) in PTDM vs. non-PTDM patients. ⮚ The presence of metabolic syndrome—an independent risk factor for PTDM (OR 1.28, 95% CI 1.04–1.51, *p* = 0.038). ⮚ FPG > 5.6 mmol/L and BMI > 28 kg/m^2^ (obesity)—predictors of PTDM. Conclusions: Presence of metabolic syndrome and its components are independent risk factors for PTDM in Chinese nondiabetic patients receiving a first renal transplant. Interventions aimed at improving pretransplant metabolic syndrome may reduce the incidence of PTDM.	A	[105]
Retrospective study	828 Caucasian renal transplant recipients	⮚ Independent risk factors for PTDM: low-grade (<1 g/day) (HR: 2.04 (1.25–3.33), *p* = 0.0042) and very low-grade (<0.3 g/day) (HR: 2.21 (1.32–3.70), *p* = 0.0025) proteinuria. Dose-dependent effect. ⮚ Tacrolimus, sirolimus and beta-blockers (HR: 1.86 (1.07–3.22), *p* = 0.0277) were significantly associated with PTDM. ⮚ Systolic arterial pressure (HR per 10 mmHg: 1.16 (1.03–1.29), *p* = 0.0126) and pulse pressure (HR: 1.26 (1.12–1.43), *p* = 0.0002) were associated with PTDM.	A	[106]
Comparative study	199 nondiabetic patients (128 men; age: 53 ± 11 years; body mass index (BMI) 24.98 ± 3.76 kg/m^2^); 45 developed PTDM	⮚ Greater BMI (*p* = 0.005), lower adiponectin levels (*p* < 0.001) and higher CRP (*p* = 0.032) in PTDM patients. ⮚ Calcineurin inhibitor, pretransplant BMI and adiponectin—predictors of PTDM. ⮚ Adiponectin concentration of 11.4 μg/mL had a significant negative prediction for PTDM risk (sensitivity: 81% and specificity: 70%). Conclusions: Adiponectin proved to be an independent predictor of NODAT.	S	[109]
Systematic study	526 kidney transplant recipients; 16.7% of patients developed PTDM	⮚ Risk factors: higher age, body mass index (BMI). ⮚ Acute cellular rejections—most relevant risk factor (HR 3.7). ⮚ Antirejective treatment with steroid pulses and conversion to tacrolimus—factor with the highest relative risk for the onset of PTDM (RR 3.5). Conclusions: Based upon a higher rate of acute rejections (AR), the necessity of frequent antirejective treatments was more relevant for the induction of PTDM than age or BMI.		[111]
Pathophysiology				
In vivo/animal study	Male and female Sprague-Dawley rats receiving TAC, SIR, TAC and SIR, or control for 2 weeks. All rats were administered an oral glucose challenge at the end of treatment	⮚ β-cell mass was reduced significantly after TAC treatment in male rats. ⮚ SIR did not affect β-cell mass, regardless of sex. ⮚ Conclusions: SIR impairs insulin signaling, without any effect on β-cell mass, and TAC does not impair insulin signaling but reduces β-cell mass.	A	[44]
In vitro	26 pancreas allograft biopsies, performed 1–8 months post-transplantation, from 20 simultaneous kidney-pancreas transplant recipients, randomized to receive either TAC or CSA	⮚ The islet cell damage was more frequent and severe in the group receiving TCA than in the group receiving CSA. ⮚ Association between toxic levels of CSA or TCA and concurrent administration of pulse steroids and hyperglycemia (*p* = 0.005). ⮚ Cytoplasmic swelling and vacuolization, and marked decrease or absence of dense-core secretory granules in βcells were more pronounced in patients on TAC. Conclusions: Structural damage to βcells can at least partially account for the glucose metabolism abnormalities seen in patients receiving these drugs. Toxic levels of CSA or TAC and higher steroid doses potentiate each other’s diabetogenic effects.	S	[45]
In vitro/In vivo###	Human islets/rat islets/ INS-1 rat insulinoma cells/male C57BL/6 mice	⮚ Significantly increased human β-cell apoptosis following treatment with calcineurin inhibitor tacrolimus. ⮚ Tacrolimus significantly decreased rodent β-cell replication, but not human β-cell replication. ⮚ Tacrolimus decreased Akt phosphorylation, suggesting that calcineurin could regulate replication and survival via the PI3K/Akt pathway. ⮚ Insulin receptor substrate-2 (Irs2)—a novel calcineurin target in β-cells. ⮚ Irs2 mRNA and protein are decreased by calcineurin inhibition in both rodent and human islets. ⮚ The effect of calcineurin on Irs2 expression is mediated at least in part through the nuclear factor of activated T-cells (NFAT). Conclusions: Calcineurin is a regulator of human β-cell survival in part through the regulation of Irs2.	S	[46]
Predefined substudy of a previously published randomized trial	Renal transplant recipients on CNI treatment (*n* = 23) vs. CNI-avoidance (*n* = 21)	⮚ Insulin sensitivity was significantly better after 12 months in patients never treated with CNI drugs (0.091 (0.050) vs. 0.083 (0.036) μmol/kg/min/pmol/L, *p* = 0.043). ⮚ Insulin secretion tended to be higher in CNI treated patients (*p* = 0.068). Conclusions: Long-term CNI treatment negatively affects glucose metabolism.	A	[47]
Prospective study	26 kidney transplant patients who discontinued CSA to take sirolimus, 15 recipients of suboptimal kidneys treated with tacrolimus + sirolimus for the first 3 mo after grafting and then with sirolimus alone	⮚ Withdrawal of CSA or tacrolimus was associated with a significant fall of insulin sensitivity (both *p* = 0.01) and with a defect in the compensatory βcell response (*p* = 0.004 and *p* = 0.02, respectively). ⮚ This increase of insulin resistance and the decrease of disposition index significantly correlated with the change of serum triglyceride concentration (R(2) = 0.30, *p* = 0.0002; and R(2) = 0.19, *p* = 0.004, respectively). Conclusions: Discontinuation of calcineurin inhibitors and their replacement by sirolimus fail to ameliorate the glycometabolic profile of kidney transplant recipients.	S	[57]
Retrospective study	146 renal transplant recipients	⮚ Significantly lower cumulative prevalence of IFG and PTDM 30-months post-transplantation in patients switched to an immunosuppression with EVR compared to patients on continued CSA treatment (10% vs. 22%, *p* = 0.049). ⮚ Patients switched to EVR showed a higher incidence of acute cellular rejections in the first 12 months (23% vs. 11%, *p* = 0.048). Conclusions: EVR-based immunosuppression was associated with a similar or even improved glycemic control and improved renal function, but also with higher rejection rates.	S	[58]
Analysis of two randomized, multicenter trials	Kidney transplant recipients switched (at month 4.5) to everolimus, receiving standard cyclosporine (CsA)-based regimen (ZEUS, *n* = 300), or switched (at month 3) to everolimus, remaining on standard CNI therapy or convert to everolimus with reduced-exposure CsA (HERAKLES, *n* = 497)	⮚ No difference in the incidence or severity of PTDM with early conversion from a CsA-based regimen to everolimus, or in the progression of pre-existing diabetes.	S	[59]
In vitro study	MIN-6 insulinoma cells	⮚ Rapamycin had a dose-dependent, time-dependent, and glucose-independent deleterious effect on MIN-6 cell viability. ⮚ Furthermore, 10 and 100 nmol/L rapamycin caused apoptosis in MIN-6 cells. Conclusions: A supra-therapeutic rapamycin concentration of 100 nmol/L significantly impaired glucose- and carbachol-stimulated insulin secretion in rat islets and had a deleterious effect on the viability of rat and human islets, causing apoptosis of both α- and β-cells.	A	[60]

BMI—body mass index; BPAR—biopsy-proven acute rejection; Cl—confidence interval; CNI—calcineurin inhibitors; CSA—cyclosporine; EVR—everolimus; FPG—fasting plasma glucose; HR—hazard ratio; IFG—impaired fasting glucose; OR—odds ratio; PTDM—post-transplant diabetes mellitus; RTR—renal transplant recipients; S—steroids; SIR—sirolimus; TAC—tacrolimus.

**Table 2:** The summary of results of studies concerning the diagnosis as management of PTDM.

Population and Study Design	Result	References
Single-center, unblinded, pilot randomized controlled trial (19 patients) assessing the feasibility, tolerability and efficacy of metformin after renal transplantation in patients with impaired glucose tolerance (IGT)	⮚ Using OGTT at 1 year as an end point for efficacy would be reasonable as it remains the gold standard for PTDM diagnosis. ⮚ The use of metformin in renal transplant recipients with IGT appeared safe and had good tolerability with no serious adverse events.	[1,162]
191 kidney transplants who had at least 1-year follow-up post-transplant	⮚ San Antonio Diabetes Prediction Model (SADPM) and Framingham Offspring Study–Diabetes Mellitus (FOS-DM) algorithm can be used to identify kidney recipients at higher risk for PTDM beyond the first year. ⮚ SADPM score detects some 25% of kidney transplant patients with an eightfold risk for PTDM.	[148]
Randomly assigned 3234 nondiabetic persons with elevated fasting and post-load plasma glucose concentrations to placebo, metformin, or a lifestyle-modification program	⮚ Lifestyle changes and treatment with metformin both reduced the incidence of diabetes in persons at high risk. ⮚ The lifestyle intervention was more effective than metformin.	[149]
Randomly assigned 522 middle-aged, overweight subjects (172 men and 350 women) with impaired glucose tolerance to either the intervention group (individualized counselling aimed at reducing weight, total intake of fat, and intake of saturated fat and increasing intake of fiber and physical activity) or the control group	⮚ The risk of diabetes was reduced by 58% (*p* < 0.001) in the intervention group. ⮚ The reduction in the incidence of diabetes was directly associated with changes in lifestyle. ⮚ Type 2 diabetes can be prevented by changes in the lifestyles of high-risk subjects.	[150]
Randomized controlled trial of conventional policy, primarily with diet alone (*n* = 411) versus intensive blood-glucose control policy with metformin in 753 patients recruited to UKPDS in 15 centers	⮚ Patients allocated metformin, compared with the conventional group, had risk reductions of 32% (95% CI 13–47, *p* = 0.002) for any diabetes-related endpoint, 42% for diabetes-related death (9–63, *p* = 0.017), and 36% for all-cause mortality (9–55, *p* = 0.011). ⮚ Among patients allocated intensive blood-glucose control, metformin showed a greater effect than chlorpropamide, glibenclamide, or insulin for any diabetes-related endpoint (*p* = 0.0034), all-cause mortality (*p* = 0.021), and stroke (*p* = 0.032). ⮚ Intense glucose control with metformin appears to decrease the risk of diabetes-related endpoints in overweight diabetic patients.	[154]
154 consecutive patients with a body mass index ≥27 kg/m^2^	⮚ Metformin is an effective drug to reduce weight in a naturalistic outpatient setting in insulin sensitive and insulin resistant overweight and obese patients.	[155]
138 patients on active kidney transplant waiting list at the Tübingen University Hospital Collaborative Transplant Center	⮚ Among waiting list patients, disturbances in glucose metabolism are substantially higher than previously anticipated. ⮚ Complete characterization of glucose metabolism is mandatory to identify patients at risk for progression to DM or PTDM, since the diagnostic accuracy of HbA1c alone in chronic kidney disease (CKD) and dialysis patients is limited. ⮚ Impaired insulin sensitivity is compensated in part by increased insulin secretion, weakening correlation of BMI to clinical endpoints—the risk of prediabetes increases with age and BMI. ⮚ Early identification of patients at risk and knowledge of underlying pathomechanisms may also help tailoring immunosuppression which is a major modifiable variable for glycemic control, as for the risk of allograft rejection, infection and malignancy. ⮚ Selected patients with insulin resistance, i.e., overweight patients, may benefit from steroid-free maintenance immunosuppression, as corticosteroids negatively affect insulin sensitivity.	[20]

## Data Availability

Not applicable.
